# Brain organoid protocols and limitations

**DOI:** 10.3389/fncel.2024.1351734

**Published:** 2024-03-20

**Authors:** Helen H. Zhao, Gabriel Haddad

**Affiliations:** ^1^Department of Pediatrics, University of California, San Diego, La Jolla, CA, United States; ^2^Department of Neurosciences, University of California, San Diego, La Jolla, CA, United States; ^3^The Rady Children's Hospital, San Diego, CA, United States

**Keywords:** cerebral organoid, protocol, limitations, stem cell, human diseases

## Abstract

Stem cell-derived organoid technology is a powerful tool that revolutionizes the field of biomedical research and extends the scope of our understanding of human biology and diseases. Brain organoids especially open an opportunity for human brain research and modeling many human neurological diseases, which have lagged due to the inaccessibility of human brain samples and lack of similarity with other animal models. Brain organoids can be generated through various protocols and mimic whole brain or region-specific. To provide an overview of brain organoid technology, we summarize currently available protocols and list several factors to consider before choosing protocols. We also outline the limitations of current protocols and challenges that need to be solved in future investigation of brain development and pathobiology.

## Definition of organoids

Organoid refers to a mini cluster of cells growing in a three-dimensional (3D) environment *in vitro* and recapitulating a structural and functional organ *in vivo* (Corro et al., 2020). Human organoids can be formed from tissue fragments or stem cells because cells and tissues can self-organize and dissociate-aggregate. Organoids derived from human tissue samples, such as biopsies, surgical specimens, or fetal material, are also called primary organoids (Cala et al., 2023). Most primary organoids use extracellular matrix (ECM) as scaffolds to support the growth of primary cells under specific culture conditions. For example, breast epithelial cells can fully form 3D ducts and ductules that exhibit the function of milk protein secretion by using matrigel as scaffolds (Li et al., 1987; Shannon et al., 1987; Dekkers et al., 2021). With the rapid development of stem cell technologies, 3D organoids derived from stem cells have been widely adopted; many studies have been using stem cells derived organoids to recapitulate the key structure and function of organs such as the kidney, lung, intestine, brain, and retina under physical or pathological conditions (Lancaster et al., 2013; Morizane et al., 2015; Crespo et al., 2017; Miller et al., 2019; Norrie et al., 2021).

## Generation of brain organoids

Due to the inaccessibility of human brain tissue and lack of appropriate *in vitro* models, our understanding of human brain development and function still needs to catch up. Recent advances in 3D organoid technology provide us with a powerful tool to investigate the complexity of brain development and functions. The first study of 3D cerebral organoids was published by Lancaster et al. (2013). Since then, many brain organoid protocols have been developed (Qian et al., 2018; Sloan et al., 2018; Tanaka et al., 2020; Kim et al., 2021; Valiulahi et al., 2021; Lee et al., 2022). In the current review, 223 articles were obtained from Pubmed using the search terms “protocol, brain organoids, stem cell” from 2013 to 2023; we removed reviews and brain organoid-unrelated articles and added a few articles through cross-referencing. A total of 114 articles were included and reviewed in Supplementary Table 1.

**Figure 1:**
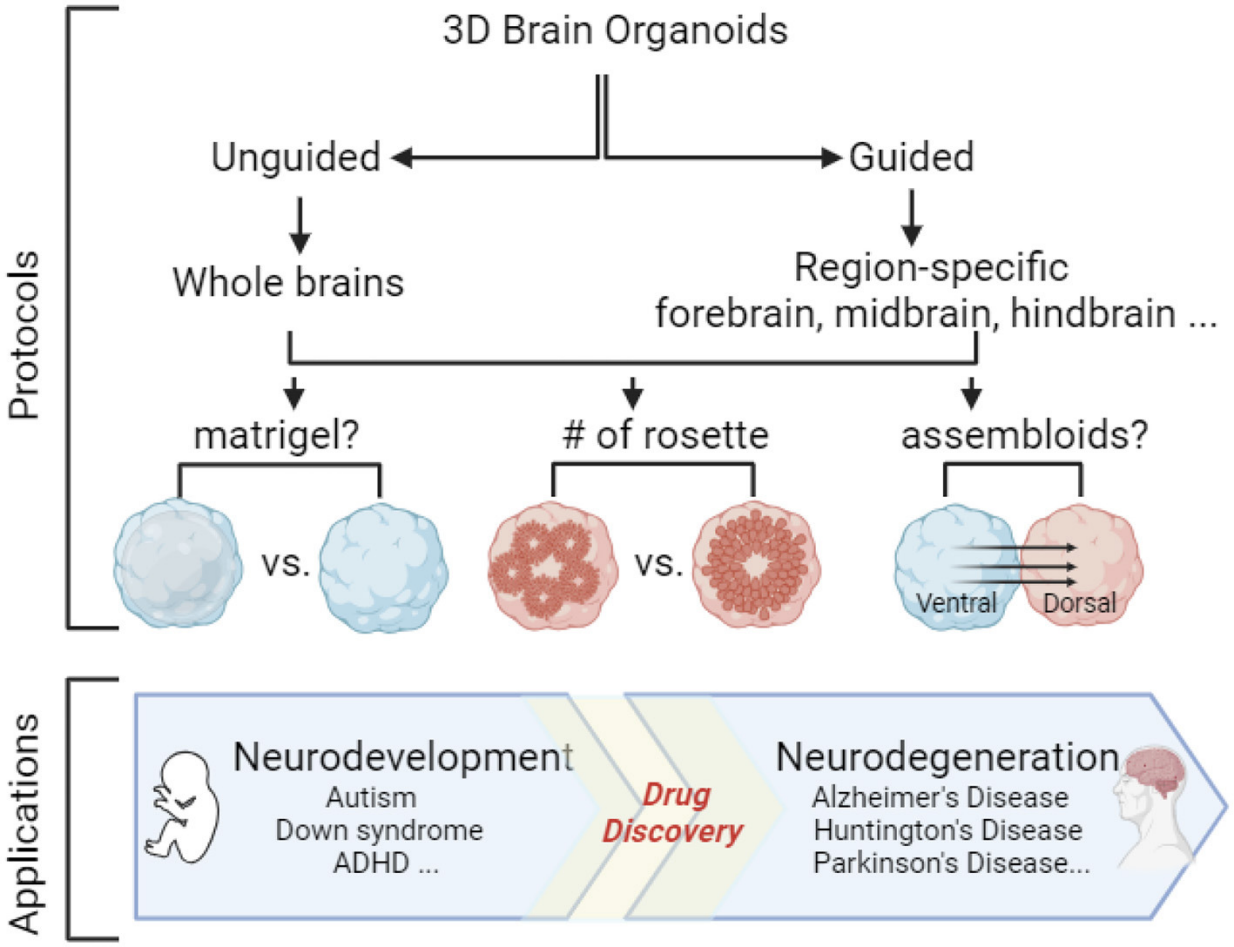
3D Brain organoids can be generated through guided and unguided protocols to mimic either the whole brain or specific brain regions. Several factors such as the use of extracellular matrix (e.g., matrigel), the number of rosettes within each organoid, and the choice of assembloids should be considered before deciding on the protocol. 3D organoids can be used as a model system to investigate neurodevelopmental, and neurodegenerative diseases, as well as drug discovery. The figure was created with Biorender.com.

*Organoid protocol*: Generation of brain organoids starts with 3D embryoid body (EB) formation, neural induction, differentiation, and maturation. Brain organoids can be generated through either unguided (36 out of 114 articles) or guided (78 out of 114 articles) protocols. Through the unguided protocol, stem cells undergo spontaneous differentiation without any extrinsic factors, and these organoids contain multiple cell types and brain regions (Lancaster et al., 2013; Lancaster and Knoblich, 2014). In contrast, stem cells undergo guided differentiation (Eiraku et al., 2008; Kadoshima et al., 2013; Pasca et al., 2015) by the addition of extrinsic factors to mimic morphogen gradient during embryonic brain development and to pattern these organoids with region-specific identity. Patterning of region-specific organoids is achieved by manipulations of Suppressor of Mothers Against Decapentaplegic (SMAD), Wingless/integrated (WNT), Sonic hedgehog (SHH), retinoic acid (RA), and other signaling pathways, such as FGF and Notch, during EB formation and neural induction. First, SMAD inhibition includes inhibiting bone morphogenetic protein (BMP) and tumor growth factor ß (TGFß) signaling pathways that promote neuroectodermal fate. Then, the dorsal and ventral patterning are obtained through BMP/WNT inhibition and SHH activation, respectively, while rostralization and caudalization are obtained by inhibition or activation of RA, WNT, and FGF signaling pathways (Tanaka and Park, 2021; Zhang et al., 2022). FGFs and Notch signals affect neuroepithelial patterning either directly or through modulations of SHH signaling pathway (Gutin et al., 2006; Kong et al., 2015; Farreny et al., 2018). Furthermore, the strength and exposure time of diffusible morphogens and crosstalk among different signaling pathways are also critical for precise pattern formation (Fattah et al., 2023). Various combinations of morphogens from each signaling pathway were applied to generate region-specific organoids, such as dorsal (Pasca et al., 2015; Sebastian et al., 2023), ventral (Sloan et al., 2018; Eigenhuis et al., 2023; Mulder et al., 2023), hippocampal (Jacob et al., 2020), cerebellum (Silva et al., 2020; Atamian et al., 2024), hindbrain (Valiulahi et al., 2021), and spinal cord (Lee et al., 2022) brain regions. As shown in Supplementary Table 1, cortical and dorsal forebrain organoids often use BMP and TGF inhibitors; in contrast, caudal parts of the brain, such as hindbrain and spinal cord organoids, frequently use WNT, RA, and FGF activators instead. Within region-specific organoid protocols, cortical organoids are the most pursued. Therefore, we use cortical organoids as an example to discuss several factors that need to be considered before deciding on protocol, such as the use of extracellular matrix (ECM), assembloids, cellular stress, and multi-rosette vs. single-rosette organoids (Figure 1).

*Extracellular matrix*: ECM is known to play an essential role in directing cell fate and differentiation, especially in promoting the stem cell niche for self-organization, differentiation, and organoid expansion (Hughes et al., 2010; Tang et al., 2023). Indeed, early exposure to exogenous ECM such as matrigel can trigger quick neuroepithelial morphogenesis (Martins-Costa et al., 2023); organoids without ECM form compact unpolarized tissues with a lack of large ventricles and loss of certain types of radial glial cells during neuronal differentiations as compared to organoids with ECM (Chiaradia et al., 2023). However, Martins-Costa et al. (2023) demonstrated that cell types and tissue morphology over long-term organoid development are comparable and independent of using exogenous ECM. Furthermore, they speculated that permanently supplementing exogenous ECM may contribute to mispatterning in the organoid culture.

ECM embedding was widely used in organoid protocols by many investigators (67 out of 114 articles). The current ECMs used in the cortical organoid culture (Heo et al., 2022) include natural scaffolds such as matrigel (Lancaster et al., 2013), decellularized tissue-derived scaffolds (Cho et al., 2021; Simsa et al., 2021) and synthetic polymer-based scaffolds (Lancaster et al., 2017; Oksdath et al., 2018; Hofer and Lutolf, 2021). Matrigel is the most used ECM preparation in the organoid protocols (Mulder et al., 2023). Matrigel is extracted from murine Engelbreth-Holm-Swarm sarcomas cells containing more than 1800 unique proteins (Hughes et al., 2010). Undefined features, manual embedding, and potential matrigel batch-to-batch variability resulted in a higher variability and lower reproducibility in organoids generated with matrigel. Although this variability can be minimized by more defined matrigel alternatives such as synthetic hydrogel scaffolds (Table 1) (Barry et al., 2017; Heo et al., 2022), the development of engineered scaffolds is still in the early stage, and the effect of these biomaterials for the organoid cultures need further investigation.

**Table 1:** Limitations of current organoid protocols.

**Limitations**	**Potential solutions**	**References**
Organoids do not fully resemble brain cytoarchitecture and function	Unguided protocol	Lancaster and Knoblich, 2014
	Assembloids	Sun et al., 2023
Variation among individual organoids or batches	Omitted matrigel embedding	Camp et al., 2015
	Use defined matrices: synthetic hydrogels	Barry et al., 2017
	Guided protocol with defined culture conditions	Renner et al., 2020
	Single rosette organoid	Knight et al., 2018
Unpredictable number of rosettes within each organoid	Single rosette organoid	Knight et al., 2018
		Wang et al., 2022
Necrotic core due to cellular stress	Bioreactor or orbital shaking	Lancaster and Knoblich, 2014
	High O2	Kadoshima et al., 2013
	Millifludic culture	Berger et al., 2018
	Sliced organoids	Qian et al., 2020
	CEPT cocktail	Ryu et al., 2023
	Transplantation	Cao et al., 2023
	Removing cell stress data from RNAseq dataset	Vertesy et al., 2022
Lack of maturation	Long term culture	Gordon et al., 2021
	Volumetric compression	Tang et al., 2023
	Microfluidic device	Cho et al., 2021
	Assembloids	Miura et al., 2022
	Transplantation	Wang et al., 2023

*Assembloids*: The development of organoid technology has enabled us to aim at assembling various regions of the brain to model human diseases *in vitro*. Like other organs, the human brain contains multiple regions and cell types. Organoids generated through the unguided protocol contain diverse cells and brain regions but can exhibit significant variations among organoids or batches (Camp et al., 2015). In contrast, region-specific organoids generated through the guided protocol contain a limited type of neurons and increased reproducibility (Chiaradia et al., 2023). Assembloids provide a partial solution to overcome such limitations (Table 1). Assembloids are obtained by incorporating two or more organoids with different regional identities into multi-region assembloids, or organoids with varying types of cell into multi-lineage assembloids, or inter-individual assembloids as well as inter-species assembloids (Pasca et al., 2022).

The human brain has three primary regions: the cerebrum, cerebellum, and brain stem. The cerebrum is further divided into two hemispheres, each with four lobes: frontal, temporal, parietal, and occipital. Each lobe can then be separated into multiple areas, and each serving distinct functions. The wiring among these areas connects the brain into a network that allows the brain to perform more sophisticated functions. The availability of region-specific brain organoid protocol will enable researchers to build a variety of assembloids to model the interconnection among these regions and mimic as much as possible developmental processes and diseases that span multiple areas of the brain (Kelley and Pasca, 2022). For instance, fusion between dorsal and ventral forebrain organoids is used to model interneuron migration (Bagley et al., 2017; Birey et al., 2017; Samarasinghe et al., 2021). Fusions of cortical organoids with other brain regions such as (1) thalamus to model thalamus dysfunction-related psychiatric disorders (Xiang et al., 2019, 2020; Angulo Salavarria et al., 2023); (2) thalamus and retina to model the projections of visual system (Fligor et al., 2021); (3) striatum to model the dysfunction in neural circuits of cortico-striatal pathway as observed in autism spectrum disorder and schizophrenia (Miura et al., 2020, 2022); and (4) spinal cord and muscle to model cortical control of motor contractions (Knecht et al., 2019; Andersen et al., 2020). Other brain regions, such as the hypothalamus and pituitary, can be fused as assembloids to model the hypothalamic-pituitary axis (Kasai et al., 2020).

The central nervous system contains neurons and non-neuronal cells such as astrocytes, oligodendrocytes, microglia, epithelial cells, pericytes, and endothelial cells. Co-culturing microglia or endothelial cells with brain organoids forms multi-lineage assembloids to study the interactions between neurons and other non-neuronal cell types that impact neuronal functions. Microglia are primary immune cells in the brain that not only protect the brain from pathological insults (Xu et al., 2016) but also play a critical role in neural development, synaptic formation, and neural network maturation (Tong and Vidyadaran, 2016; Coomey et al., 2020). Since microglia are derived from mesoderm lineage and neurons are derived from ectoderm lineage, co-cultured microglia with brain organoids can be achieved by using the unguided protocol for spontaneous formation of microglia, or introducing primary microglia and stem cell-derived microglia or microglial progenitor cells to brain organoids (Ormel et al., 2018; Zhang W. et al., 2023). Stem cell-derived microglia function and morphologically resemble *in vivo* microglia; they become ameboid and phagocytic upon activation (Haenseler et al., 2017). A controllable proportion and distribution of microglia in organoids are essential to study the microglial function under physiological conditions or to model microglial dysfunction-related neurological disorders from neuropsychiatric disorders to neurodegenerative diseases (Xu et al., 2021; Schafer et al., 2023). Co-culture of endothelial cells with brain organoids forms a vascular-like network and vascularized organoids (Pham et al., 2018; Cakir et al., 2019; Shi et al., 2020; Sun et al., 2022, 2023) which makes it possible to recapitulate the critical processes of vasculogenesis, angiogenesis, and vasculopathy related human diseases. Additionally, co-culturing peripheral neurons, such as enteric neurons, with human intestinal organoids expands our scope to study the connection between the gut and brain (Ryan et al., 1985; Hampton, 2017; Schlieve et al., 2017; Horvath et al., 2023).

*Cellular stress*: Because of a lack of functional blood vessels and other supporting non-neuronal cells, organoids cannot fully mimic the intrinsic complexity of brain tissue. Indeed, Bhaduri et al. (2020) have demonstrated that organoids across all protocols have increased activation of glycolysis and ER stress pathways and impaired cell-subtype specifications compared to fetal tissue. Increasing cellular stress and forming a necrotic core have been reported in organoids that are larger than 500 μm in diameter due to insufficient perfusion of oxygen, nutrients, and catabolites (Hirschhaeuser et al., 2010; Langan et al., 2016; Magliaro et al., 2019).

Several strategies have been attempted to address these questions (Table 1). For example, spinning bioreactor or orbital shaking instead of static culture is commonly used to improve the delivery of oxygen and nutrients in organoids (Lancaster and Knoblich, 2014). Higher levels of oxygen (40% instead of 20% O_2_) culture and continuous laminar flow supplied with the Millifludic culture system are also applied for long-term organoid culture (Kadoshima et al., 2013; Berger et al., 2018). Neocortical organoid slices cultured on an air-liquid interface allow sliced cortical plates to continue expansion, neurogenesis, and maturation (Qian et al., 2020; Giandomenico et al., 2021). Furthermore, using a small molecule cocktail named CEPT (chroman 1, emricasan, polyamines, trans-ISRIB), a polypharmacological approach can enhance cytoprotection and improve organoid survival (Ryu et al., 2023). In addition, organoid transplantation was confirmed to alleviate the cellular stress in organoids (Bhaduri et al., 2020) and improve organoid maturity, cellular complexity, and brain function (Cao et al., 2023; Jgamadze et al., 2023; Wang et al., 2023). Lastly, Vertesy et al. (2022) developed a computational algorithm method, Gruffi, to remove the stressed cells from the organoid single-cell RNAseq dataset, and therefore, improves the bioinformatic data analysis after the organoids are collected.

*Multi- or single-rosette organoids*: Although the diverse cell types and regions contribute to the complexity of the human brain, brain regions arise from one neural tube during embryogenesis *in vivo*. The current brain organoid protocols (Lancaster et al., 2013; Pasca et al., 2015) have multiple rosettes within each organoid, and each rosette acts as an independent organizing center. The unpredictable number and organization of rosettes within each organoid results in a lack of reproducibility and fidelity (Table 1). Therefore, the self-organizing single rosette (SOSR) organoid was developed and adopted by several laboratories in the past few years (Knight et al., 2018; Wang et al., 2022; Takla et al., 2023). These well-defined SOSR organoids with reproducible size and cytoarchitecture offer improved reproducibility and fidelity (Knight et al., 2018; Tidball et al., 2023) than the existing organoid protocols (Lancaster et al., 2013; Pasca et al., 2015) and can be used as a reliable model to recapitulate the neural development and related disorders in the human brain.

*In vitro organoids vs. in vivo fetal brain*: The 3D organoids recapitulate many unique features of the human brain and have been widely used as a model system to study brain development and neuropathological disorders. However, how well do *in vitro* organoids match up with the *in vivo* fetal brain condition? The equivalent age still needs to be determined.

Transcriptomic analysis is the most used method to address this question. Amiri et al. (2018) compared the transcriptomic data between fetal cortex samples and 11 to 41-day-old dorsal forebrain organoids generated through Mariani's guided protocol. They revealed that the organoids' transcriptomes map to the human fetal cortex between 8 and 16 post-conception weeks (PCW) of development (Mariani et al., 2015). To minimize the effect of organoid protocols on data analysis, Magni et al. (2022) generated the cortical organoids with three different protocols: one unguided approach with ECM embedding, two guided approaches with or without ECM embedding and WNT activation. They concluded that 3 months of cortical organoids closely resembled 9 PCW or 20 PCW human embryonic cortex samples more than 25-day early organoids (Magni et al., 2022) based on the neuronal maturation gene expression profile. Gordon et al. (2021) compared gene expression profiles of cortical organoid culture with the BrainSpan dataset at multiple time points over a more extended period (up to 652 days). BrainSpan is a reference database that contains developmental and postnatal transcriptome information from *in vivo* human brains. They reported that cortical organoids before 250 days map to the prenatal stage (10–38 PCW), and 250 to 300-day-old organoids represent the transition between prenatal and postnatal stages *in vivo* (Kang et al., 2011; Gordon et al., 2021). Recently, Cheroni et al. (2022) compared the RNAseq dataset from in-house generated cortical brain organoids (Pasca et al., 2015) and three other organoids, including forebrain organoids (Qian et al., 2016), telencephalic organoids (Mariani et al., 2015), and minimally-guided organoids (Luo et al., 2016) at comparable time-point (from 1 to 200 days) with fetal cortex at 8–37 PCW. They demonstrated that forebrain and minimally-guided organoids showed a more rapid transcriptional maturation than cortical brain organoids because 60-day forebrain and minimally-guided organoids showed a similarity with late PCE fetal cortex that cortical brain organoids reached by 100 days (Cheroni et al., 2022). Those studies further confirmed the presence of heterochronicity across different protocols when dissecting the equivalent age of brain organoids relative to the fetal brain.

Using DNA methylation sequencing, Luo et al. (2016) demonstrated that 40 to 60-day-old organoids recapitulate many epigenetic signatures of the mid-fetal (12–16 PCW) brain. Trevino et al. (2020) used the ATAC-seq to show that 40 to 80-day-old organoids resemble the human fetal brain at 8–10 PCW, 80–250-day-old organoids resemble mid- to the late fetal stage (10 PCW to birth), and postnatal stage are more similar to organoids after 350 days (Lewis et al., 2021).

To our knowledge, only one electrophysiological functional study compared the age of organoids with *in vivo* fetal brains. Trujillo et al. (2019) used multi-electrode array to record organoids for up to 10 months; they compared the local field potential of organoids with the previously published preterm EEG recordings from preterm infants ranging from 24 to 38 weeks post-menstrual age. They report that complex oscillatory waves of cortical organoids after 28 weeks of maturation resemble the electrophysiological signature of preterm human infant EEG (Trujillo et al., 2019).

In summary, 2~3-month organoid culture can mimic *in vivo* early to mid-fetal brain development, and ~10-month-old organoids are more likely to reach the transition stage between prenatal and postnatal brain development. The exact age equivalent is still challenging because different organoid protocols were used in each study. For studies that mimic drug treatment or hormone surge at a specific time or period during fetal brain development (Madhavan et al., 2018; Kelava et al., 2022), carefully choosing organoid protocol and adopting equivalent age is needed.

## Modeling human diseases using organoids

Stem cell-derived 3D organoids, especially brain organoids, have been a powerful tool to open an opportunity for human brain research and model many neurological diseases such as neurodevelopmental disorders and neurodegenerative diseases (Silbereis et al., 2016) (Figure 1).

*Modeling neurodevelopmental disorders*: Organoid culture resembles early fetal brain development and can model a variety of neurodevelopmental disorders such as autism spectrum disorders, schizophrenia, attention-deficit/hyperactivity disorder, Down syndrome, and fragile X syndrome (Chan et al., 2020; Kang et al., 2021; Notaras et al., 2022; Rabeling and Goolam, 2022; Zhao and Haddad, 2022; Santos et al., 2023; Zhang D. et al., 2023). Neurodevelopmental disorders are a group of conditions that affect the development and maturation of the human brain and impact patients' ability to learn, speech, behavior, memory, and emotions. Autism spectrum disorder (ASD) is one of the most studied neurodevelopmental disorders using stem cells and organoids (Chan et al., 2020; Santos et al., 2023). ASD has a broad spectrum of phenotypes with highly heterogeneous features. More than 1,000 genes have been reported to be associated with the risk of ASD (Antaki et al., 2022; Qiu et al., 2022). Brain organoids allow us to investigate the role of individual ASD risk genes such as FOXG1, SHNAK3, and CNTNAP2 in the neuropathology of ASD and study the potential interaction between ASD risk genes and environments or drugs *in vitro* (Mariani et al., 2015; De Jong et al., 2021; Schmidt, 2021; Meng et al., 2022; Wang et al., 2022). Furthermore, network analysis reveals that those ASD risk genes have tissue-specific transcriptional convergence implicating fetal brain development, neurogenesis, and synaptic processes (De La Torre-Ubieta et al., 2016; Wen et al., 2016; Sullivan et al., 2019; Paulsen et al., 2022). Consistently, Paulsen et al. (2022) have reported that ASD cortical organoids indeed showed cell-type-specific developmental abnormality. As compared to control organoids, ASD organoids with three individual ASD risk genes, including SUV420H1, ARID1B, and CHD8, converge on a phenotype of asynchronous neuronal development and abnormal circuit activity due to premature expansion of GABAergic neuron linage, but each gene works with different molecular mechanisms (Paulsen et al., 2022). Therefore, organoids act as a valuable tool that allows us to investigate brain development and disease pathology at the cellular, molecular and network levels and also bridge our knowledge gap between genetic analysis and neuropathological observations.

*Modeling environmental effects on neurological disorders*: Prenatal environmental adversities, including infectious agents, medication, and substance use, are risk factors for neurodevelopmental disorders. Adverse prenatal exposure is often associated with abnormal brain development and has cognitive consequences (Debost et al., 2017; Bolte et al., 2019; Smith, 2021). Organoids have been used to investigate the effect of infectious agents such as Zika virus (ZIKV), Severe acute respiratory syndrome coronavirus 2 (SARS-CoV-2), and Herpes simplex virus type I on brain cytotoxicity (Ramani et al., 2020; Xu and Wen, 2021; Qiao et al., 2022). A ZIKV outbreak, which increased the number of infants born with microcephaly in Brazil, led scientists to investigate the potential linkage between the virus and brain malformation. Studies have shown that ZIKV infection induces cell death of neural progenitor cells in brain organoids and reduces proliferation zones and disruption of cortical layers (Cugola et al., 2016; Garcez et al., 2016). Later, another study used organoids to demonstrate that duramycin and ivermectin have therapeutic potential for anti-ZIKV infection due to a significant reduction in the adverse effect of ZIKV infection on cortical development (Watanabe et al., 2017).

Prenatal substance exposure is increasingly becoming a significant public health concern due to its impact on women's health and child development (Hirai et al., 2021). For instance, maternal opioid use during pregnancy affects fetal brain development and causes cognitive dysfunction. These problems persist into adolescence or later, even if the mother was treated with methadone or buprenorphine. Kaltentach et al. used clinical assessment to evaluate brain development in infants at 3–36 months of age born to mothers who were opioid-dependent and treated with either buprenorphine or methadone during pregnancy. They reported that buprenorphine or methadone exposure had no deleterious effect on the usual physical and mental development of children (Oh et al., 2011). However, our laboratory has used brain organoids to investigate the impact of both buprenorphine and methadone on brain development. Dwivedi et al. (2023) have shown that methadone exposure altered transcriptional program, especially in synaptogenesis during early cortical development. Furthermore, Yao et al. (2020) found that methadone dose-dependently inhibits the growth of brain organoids and suppresses neural network activities; buprenorphine does not affect neural growth in brain organoids and had a mild suppression of network activities as compared with methadone (Yao et al., 2023). Therefore, organoid technology can help in our understanding of drug exposure as well as potential therapeutic modalities.

*Modeling neurodegenerative diseases*: Besides neurodevelopmental diseases, organoids can be a valuable tool for modeling neurodegenerative diseases such as Alzheimer's disease (AD), Parkinson's disease, and Huntington's disease (Bose et al., 2022; Bubnys and Tsai, 2022; Metzger et al., 2022). AD is characterized by two major pathological features: amyloid plaque (Amyloid ß accumulation) and neurofibrillary tangles (tau hyperphosphorylation). iPSC-derived organoids from both early-onset familial AD (FAD) and late-onset sporadic AD (SAD) patients accurately capture these neuropathological features and have an increased amyloid β peptide (Aβ), Aβ_42/40_ ratio and tau hyperphosphorylation (Chen et al., 2018; Gonzalez et al., 2018; Kuehner et al., 2021; Park et al., 2021; Bubnys and Tsai, 2022; Yanakiev et al., 2022). Progressively increased Aβ accumulation was observed in a time-dependent manner. For example, Raja et al. (2016) reported an increased Aβ accumulation from 60 days to 90 days in organoids with APP duplication. Similarly, Zhao and Haddad (2022) used Down syndrome (DS) organoids as a model to study AD pathology and reported an increased Aβ accumulation from 8 weeks organoids to 12 weeks organoids.

Recently, Arber et al. (2021) reported that FAD organoids with presenilin mutation not only resemble AD pathology but also exhibit abnormal neurogenesis, such as premature terminal differentiation of neural progenitor cells and a trend of reduced abundance of newborn neurons. Abnormal neurogenesis observed in AD organoids suggests that the pathology of AD brain may start as early as fetal brain development. In clinics, AD biomarkers such as cerebrospinal fluid Aß42 and Tau can be detected decades before the onset of AD dementia (Sperling et al., 2011; Bateman et al., 2012; Buchhave et al., 2012). Pre-tangle alterations with positive immunostaining of phosphor-tau AT8 are detected in about 12.5% of non-selected autopsy cases under 20 years of age (Braak et al., 2011). Therefore, Arendt et al. (2017) have proposed an argument for the developmental origin of AD. AD pathology may begin as early as the fetal stage or childhood; the developmental defect may not lead to disease but increase the susceptibility to disease onset with the second hit from either genetic or environmental stimulus later in life (Arendt et al., 2017). A similar assumption is also proposed for Parkinson's disease due to altered dopaminergic neurogenesis (Barlow et al., 2007; Schwamborn, 2018), which may partially explain why we observe the pathology of neurodegenerative disease in stem cell-derived brain organoids, a neurodevelopmental model.

## Limitations and future direction

Organoid technology is a powerful tool that revolutionized the field of biomedical research and extended the scope of our understanding of human biology and diseases in both breadth and depth. Brain organoids can be generated in large quantities and the application of brain organoids for high-throughput drug screening, transplantation, and toxicology therefore becomes time- and cost-efficient as compared to the use of traditional animal models (Lee et al., 2018; Renner et al., 2020; Wang et al., 2020; Dong et al., 2021; Groveman et al., 2021). However, limitations and challenges still exist (Table 1). For example, organoids do not fully resemble brain cytoarchitecture and function due to missing cell types and often brain parts and structures. Although incorporation of additional cell types, such as microglia, astrocytes, vascular tissue, and other brain regions, will improve the complexity of brain organoids, organoid protocols must also be improved to reduce the variation among individual organoids or batches and prevent organoids from cellular stress. Furthermore, extending the duration of organoid culture may better recapitulate the later stages of human brain development and the aging brain manifested in neurodegenerative disease.

## Author contributions

HZ: Conceptualization, Writing – original draft, Writing – review & editing. GH: Conceptualization, Funding acquisition, Supervision, Writing – review & editing.
